# CircLMTK2 acts as a novel tumor suppressor in gastric cancer

**DOI:** 10.1042/BSR20190363

**Published:** 2019-05-21

**Authors:** Jian He, Jie Chen, Ben Ma, Li Jiang, Guangfa Zhao

**Affiliations:** 1 Department of Gastric Surgery, Fudan University Shanghai Cancer Center, Shanghai, People’s Republic of China; 2 Department of Oncology, Shanghai Medical College, Fudan University, Shanghai, People’s Republic of China; 3 Department of Head and Neck Surgery, Fudan University Shanghai Cancer Center, Shanghai, People’s Republic of China; 4 Department of Orthopedic Surgery, Huashan Hospital, Fudan University, Shanghai, People’s Republic of China

**Keywords:** cell migration and invasion, circular RNA, gastric cancer, survival

## Abstract

Recently, circRNAs have been found to play regulatory roles in cancer. In the present study, we aimed to investigate the characteristics and effect of circLMTK2, and its potential role as a novel biomarker in cases of gastric cancer (GC). About 111 pairs of clinical tissues from patients were collected for circLMTK2 expression investigation. Afterward, the relationship of circLMTK2 expression level and clinical features, such as survival, tumor size and so on, were analyzed, along with a multivariate Cox hazards analysis. Finally, malignant biological properties, like cell viability and mobility, were explored in cell line MGC-803. We found that circLMTK2 was a stable circRNA generated from the back-spliced Exon 10 and Exon 11 of the LMTK2 gene in GC cells. CircLMTK2 expression was significantly down-regulated in gastric carcinoma tissue specimens (*P*<0.001) compared with its expression in paired normal tissues. Furthermore, a Kaplan–Meier analysis revealed that lower levels of circLMTK2 expression were associated with decreased overall survival (OS) (*P*<0.001), and a multivariate Cox hazards analysis showed that high circLMTK2 expression was an independent factor for OS. Afterward, overexpression of circLMTK2 was performed in gastric cancer cell line MGC-803, and results indicated that malignant biological properties were inhibited by circLTMK2 overexpression. The present study showed the first evidence that circLMTK2 was down-regulated in GC, suggesting it as a novel biomarker for prognosis, and also as a therapeutic target in treatment of GC.

## Introduction

Most human pre-mRNAs exist in linear forms that retain the exon order. Recently, high-throughput RNA sequencing and computational analyses have revealed that circular transcripts from genetic loci were widespread in the eukaryotic transcriptome [1,2], and a series of circular RNAs (circRNAs) were found to be abundantly and stably expressed in various tissues and across different species [3–6]. CircRNAs are characterized by their covalently closed loop structures generated from back-spliced exons, and have been identified as a novel class of diverse endogenous non-coding RNAs that might regulate gene expression in eukaryotic cells [7,8]. An increasing number of studies suggest that circRNAs are involved in the development and progression of various cancer types, and they may become potential molecular biomarkers and targets for cancer treatment.

Gastric cancer (GC) remains one of the most common cancer types in the world, and has a particularly high incidence in East Asia [9]. Although early stage GC patients stage can be cured and have a good prognosis, many patients diagnosed at an advanced stage of their disease experience only a marginally improved survival time. Therefore, it is critically important to identify novel biomarkers and prognostic indicators that reflect disease status, and then develop appropriate therapeutic plans for GC patients. The circRNA hsa_circ_0001725, also termed circLMTK2, is generated by the *LMTK2* gene, and was screened for its differential expression in GC tissues by using high-throughput RNA sequencing and a bioinformatics analysis. To better characterize the potential role of using circLMTK2 as a biomarker in cases of GC, we investigated the basic features of circLMTK2 in GC cells and the association between circLMTK2 expression with the clinical outcomes of GC patients.

## Materials and methods

### Patients and clinicopathological data

From December 2007 to December 2012, consecutive tissue specimens were gathered from 111 patients who received a D2 radical gastrectomy (R0 resection) and were pathologically diagnosed as GC at the Fudan University Shanghai Cancer Center (FUSCC). We retrospectively examined medical record data concerning each patient’s clinicopathological features, including gender, age at diagnosis, maximum tumor size, differentiation grade, lymphatic invasion, nervous tissue invasion, and TNM stage. Each patient was staged according to the TMN classification system used by the 2009 American Joint Committee on Cancer/International Union against Cancer. The tissue specimens were subjected to repeated evaluation to confirm their diagnosis based on histological characteristics. Each patient provided a written informed consent for collection of their specimens and for the information obtained to be used for purposes of research, and stored in the hospital database. The study protocol was approved by the Ethics Committee of the FUSCC. All procedures were performed in accordance with the ethical standards of our institutional research committee, as well as with the 1964 Helsinki declaration and its later amendments or comparable ethical standards.

### Human GC cell lines, cell culture, and treatments

Human-derived GEL-1, SGC-7901, MGC-803, BGC-823, AGS, and MKN45 cells were obtained from the Cell Bank of the Chinese Academy of Sciences (Shanghai, China), and cultured in Roswell Park Memorial Institute 1640 medium (Gibco, Grand Island, NY, U.S.A.) containing 10% heat-inactivated fetal bovine serum (Gibco, Grand Island, NY, U.S.A.) at 37°C in a 5% CO_2_ chamber. GEL-1 cells were obtained from normal gastric epithelium cells, and the other cell lines were derived from human GC cells. Gene transcription was blocked by the addition of 2 μg/ml actinomycin D or dimethylsulphoxide (Sigma-Aldrich, St Louis, MO, U.S.A.) to the cell culture medium.

### RNA extraction and real-time qRT-PCR analysis

The nuclear and cytoplasmic fractions of cells were extracted using NE-PER Nuclear and Cytoplasmic Extraction Reagents (Thermo Scientific, Waltham, MA, U.S.A.). The total RNA from surgical specimens and cell lines was extracted using QIAamp RNA Mini Kits (QIAGEN, Chatsworth, California, U.S.A.) according to the manufacturer’s instructions. The extracted RNA was reverse transcribed to generate cDNA that was used for the real-time quantitative reverse transcription-polymerase chain reaction (qRT-PCR) as previously described [10]. The primers for circLMTK2 were as follows: (Forward) 5′-GGAAGAAGGAAAAGAAGGC-3′; (Reverse) 5′-TGGAAAGGTTTGAATACGC-3′. ACTIN was used as a housekeeping gene. The qRT-PCR assay for each sample was performed in triplicate, and the mean value was used for calculating the mRNA expression level. The relative mRNA expression levels for circLMTK2 were determined by using the Ct (2^−∆∆Ct^) method. The level of target gene expression is expressed as a ratio compared with the level Actin expression.

### Recombinant vector construction

Sequences of circLMTK2 were synthesized by GeneScript Biotech (Nanjing, Jiangsu, China) with restriction enzyme cutting site of *BamHI* and *EcoRI*. Then, sequences were linked into circRNA expression plasmid, pcircRNA1.1 (BersinBio, Guangzhou, Guangdong, China). Recombinant vector was identified using agarose gel electrophoresis after digested followed by plasmid extraction.

### Cell-counting kit-8 assay

MGC-803 cells at density of 1 × 10^4^ cells/well were plated into 96-well plate. Then, cells were transfected with plasmid of circLMTK2 or empty vector using Lipofectamine 3000 (ThermoFisher). Afterward, plates were placed into cell incubator. Cells were collected and analysis for cell viability using Cell-counting kit-8 (Dojindo, Cat.No.CK04) at 0, 24, 48, and 72 h after transfection.

### Transwell assay

Cells transfected with empty vector or circLMTK2 were cultured in serum-free condition for 12 h. Starved cells (approximately 2 × 10^4^ cells per well) were then transferred into upper chamber maintained with 0.2% FBS cultural medium. As for cell invasion assay, a Matrigel purchased from BD (No. 356234, U.S.A.) was paved at the inner side of upper chamber. Afterward, upper chambers were obtained 48 h later, and cells attached in the inner bottom of chamber were cleaned. Finally, chambers were then stained with Crystal Violet stain solution, and migrated cells and invaded cells were observed and recorded using microscope (Olympus CX41).

### Western blot assay

Cells were lysed using RIPA lysis buffer (Beyotime biotech, Shanghai, China) for total protein extraction. Then total protein solution was quantified using BCA kit (Beyotime biotech, Shanghai, China). Primary antibodies against GAPDH (A00227-1, 0.3 μg/ml, Boster, Wuhan, China), Bax (A100183, 0.3 μg/ml, Boster, Wuhan, China), and Bcl-2 (A00040-1, 0.2 μg/ml, Boster, Wuhan, China) were used for immunoblotting, followed by incubating with secondary antibody against IgG (ab7090, 1:10000, ABCAM, Cambridge, U.S.A.). Protein signal was collected using enhanced chemiluminescence. GAPDH expression in this research was served as the internal control.

### Statistical analysis

Continuous results are expressed as the mean ± standard deviation (SD). A paired *t*-test and independent *t*-test were used to compare continuous variables in two groups. Categorical data are summarized as frequencies and percentages. To verify the associations between circLMTK2 and clinical outcomes, patients were divided into two subgroups (low expression and high expression) according to their median level of circLMTK2 expression. Moreover, multivariate analyses were performed to identify risk factors for overall survival and disease-free survival in GC. A *P*-value < 0.05 was considered to be statistically significant. All statistical analyses were performed using GraphPad Prism 6.0 and SPSS for Windows (SPSS Inc., Chicago, IL).

## Results

### Characterization of circLMTK2 in GC

**Figure 1:**
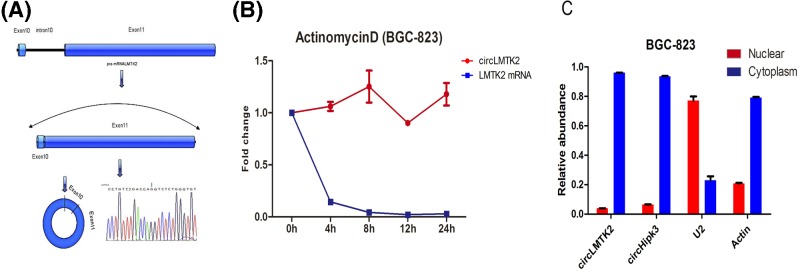
Basic features of circLMTK2 in GC (**A**) CircLMTK2 was generated from the back-spliced Exon 10 and Exon 11 of the LMTK2 gene located at chromosomal region 7q21.3. (**B**) The half-life of circRNA in actinomycin D-treated BGC-823 cells exceeded 24 h, whereas the corresponding linear transcript had a half-life of <4 h. (**C**) A qRT–PCR analysis showed that the circular form of LMTK2 preferentially localized in the cytoplasm. CircHipk3 served as a positive control.

As shown in Figure 1A, the circLMTK2 was generated from the back-spliced Exon 10 and Exon 11 of the LMTK2 gene located at chromosomal region 7q21.3. Expression of circLMTK2 was validated by RT–PCR followed by Sanger sequencing, and the splice length of circLMTK2 was 3109 nucleotides. The stability and localization of this circRNA were then investigated in BGC-823 cells that had been derived from human GC cells. A comparative analysis of circLMTK2 and LMTK2 mRNA expression was performed in BGC-823 cells that had been treated with the transcription inhibitor, actinomycin D. The analysis revealed that the circRNA was highly stable, and had a transcript half-life >24 h, whereas the corresponding linear transcript had a half-life of <4 h (Figure 1B). A distribution analysis of nuclear and cytoplasmic circLMTK2 RNA expression by qRT-PCR demonstrated that the circular form of LMTK2 preferentially localized in the cytoplasm (Figure 1C). In summary, the above results suggested that circLMTK2 was a stable circular RNA expressed in GC cells.

### Expression of circLTMK2 is down-regulated in gastric carcinoma tissues

A total of 111 patients from the FUSCC were enrolled in the present study. There were 90 males (81.10%) and 21 females (18.90%), with an age range of 19 to 79 years (mean = 59.9 years). Data concerning tumor location, size, differentiation grade, TNM stage, lymphatic invasion, and nervous invasion are provided in Table 1. The median patient follow-up time was 24 months. The tumor’s TNM stage exhibited a significant negative correlation with circLTMK2 expression (Table 1), and patients with lower levels of circLTMK2 expression had a higher incidence of lymphatic metastasis.

**Table 1:** Clinicopathological correlation of circLMTK2 expression in gastric cancer

	No.	%	High expression	Low expression	*P-*value
**Gender**					
Male	90	81.1%	45	45	
Female	21	18.9%	11	10	0.846
**Age** (years)					
≤60	52	46.8%	25	27	
>60	59	53.2%	31	28	0.642
**Tumor size** (cm)					
≤5	69	62.2%	37	32	
>5	42	37.8%	19	23	0.396
**TNM stage**					
I+II	37	33.3%	24	13	
III+IV	74	66.7%	32	42	0.032
**Lymphatic vascular invasion**					
No	52	46.8%	31	21	
Yes	59	53.2%	25	34	0.071
**Nerve infiltration**					
No	53	47.7%	28	25	
Yes	58	52.3%	28	30	0.635
**Lymphatic metastasis**					
No	28	25.2%	20	8	
Yes	83	74.8%	36	47	0.010

### Analysis of circLMTK2 expression in paired GC and para-carcinoma tissue

**Figure 2:**
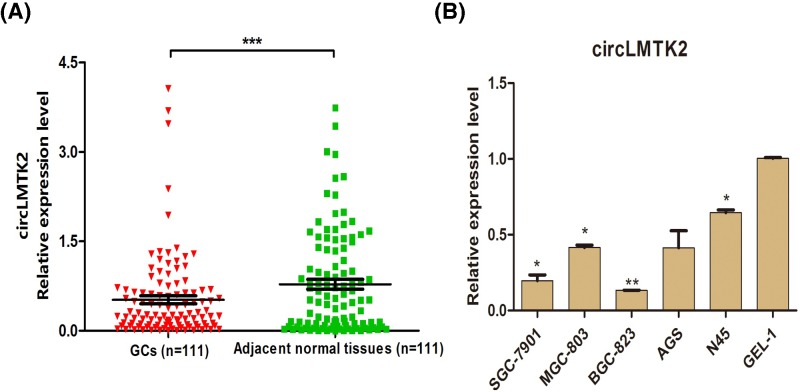
Analysis of circLMTK2 expression in paired GC and normal tissues (**A**) CircLMTK2 was expressed at significantly lower levels in GC tissues than in adjacent normal tissues. ***P<0.001 vs. adjacent normal tissues. (**B**) CircLMTK2 expression was significantly decreased in GC-derived SGC-7901 (*P*=0.030), MGC-803 (*P*=0.018), BGC-823 (*P*=0.001), and MKN45 (*P*=0.034) cells when compared with its expression in normal gastric tissue-derived GEL-1 cells. *P<0.05, **P<0.01 vs. GEL-1 cell line.

We detected the levels of circLMTK2 expression in the carcinoma specimens and paired normal tissues from 111 GC patients. When compared with circLMTK2 expression in the adjacent normal tissues, circLMTK2 was expressed at significantly lower levels in the GC tissues (*P*<0.001) (Figure 2A). Additionally, circLMTK2 expression levels were also detected in human-derived GEL-1, SGC-7901, MGC-803, BGC-823, AGS, and MKN45 cells. When compared with circLMTK2 expression in normal gastric tissue-derived GEL-1 cells, significantly decreased levels of circLMTK2 expression were found in the GC-derived SGC-7901 (*P*=0.030), MGC-803 (*P*=0.018), BGC-823 (*P*=0.001), and MKN45 (*P*=0.034) cells (Figure 2B). There was no significant difference in circLMTK2 expression between the AGS and GEL-1 cell lines (Figure 2B, *P*>0.05).

### The potential prognostic value of circLMTK2 in GC

**Figure 3:**
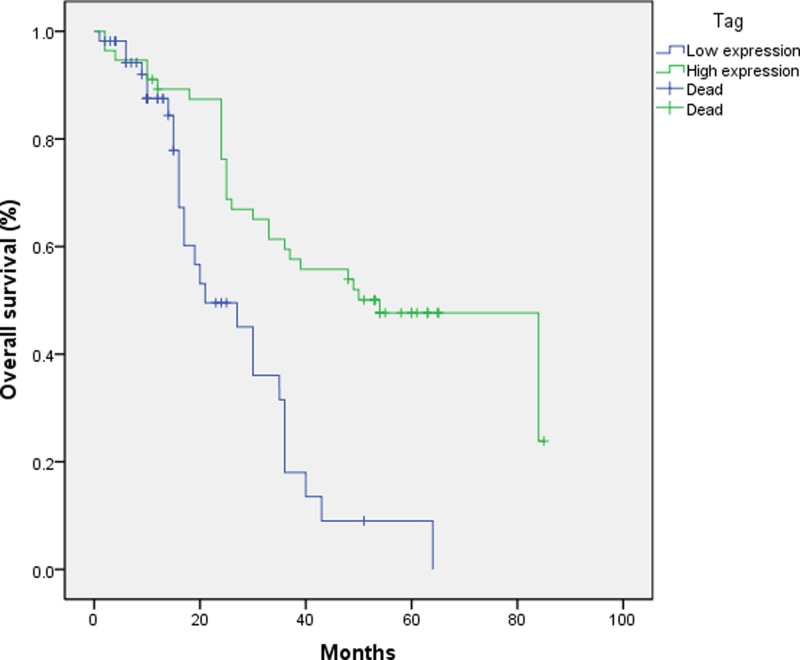
Association of circLMTK2 expression with OS A Kaplan–Meier analysis revealed that low circLMTK2 expression was associated with decreased OS (*P*<0.05, cutoff value: 0.275).

A survival analysis was performed to determine whether a patient’s level of circLMTK2 expression could be used as a prognostic factor. The median survival time of all the enrolled GC patients was 24 months (range, 1–85 months). Figure 3 shows the overall survival for GC patients with different levels of circLMTK2 expression. Patients with lower levels of circLMTK2 expression had significantly shorter survival times than patients with higher levels of circLMTK2 expression (*P*<0.05, Figure 3). A Cox regression analysis performed by multivariate analysis revealed that circLMTK2, TNM stage, and age were factors that significantly affected the prognosis of GC patients (*P*=0.004, *P*=0.009, and *P*=0.006, respectively) (Table 2). These results suggest circLMTK2 as a factor that can be used for predicting the prognosis of GC.

**Table 2:** Multivariate analysis of independent prognostic factors of CircLMTK2

Variable	Male/Female	*P-*value
Gender (Male/Female)	90/21	0.545
Age (≤60 y/≥60 y)	52/59	0.006
Tumor size (≥5 cm/≤5 cm)	69/42	0.286
TNM stage (I/II or III/IV)	37/74	0.009
Lymphatic vascular invasion (No/Yes)	52/59	0.319
Nerve infiltration (No/Yes)	53/58	0.084
Lymphatic metastasis (No/Yes)	28/83	0.693
CircLMTK2 expression (Low/High)	55/56	0.004

### Overexpression of circLMTK2 inhibited malignant biological properties

**Figure 4:**
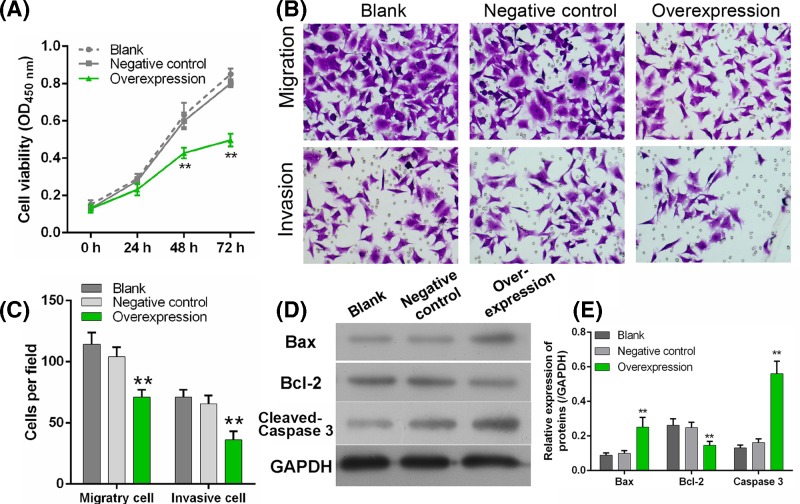
Effect of circLMTK2 on gastric cells viability, mobility (**A**) Cell viability was measured after transfected with circLMTK2 overexpressed plasmid. (**B** and **C**) Cell migration and invasion were explored using Transwell assay. (**D** and **E**) Bax, cleaved caspase 3, and Bcl-2 expression were measured using Western blot. **P<0.01 vs. Negative control. GAPDH was used as a loading control.

To explore the role of circLMTK2 in gastric carcinoma cell, MGC-803 was cultured and cell viability, Transwell assay was investigated. As shown in Figure 4, cells transfected with circLMTK2 overexpressed plasmid exhibited a significant inactive cell viability compared with negative control at 48 and 72 h (Figure 1A). Transwell assay also showed that cell mobility was significantly inhibited by circLMTK2 overexpression (Figure 4B,C). Finally, Bax and Bcl-2, a pro-apoptotic protein and proliferation protein respectively, were measured using Western blot. Results showed that Bax and cleaved Caspase 3 expression was enhanced significantly after circLMTK2 overexpression, accompanying with suppressed Bcl-2 (Figure 4D,E). These results indicated circLMTK2 can inhibit malignant biological properties by restraining proliferation, mobility, and promoting apoptosis.

## Discussion

An increasing number of studies have reported involvement of circRNAs in cancer development and progression. Additionally, circRNA ciRs-7 has been suggested as a crucial factor significantly involved in the functioning of neurons as well as neurological disorders and brain tumor development [11]. A global reduction of circular RNA abundance was observed in colorectal cancer cell lines and cancer tissues when compared with normal tissues [12]. Li et al. [13] proved that cir-ITCH exerts an inhibitory effect on esophageal squamous cell carcinoma by regulating the Wnt pathway [13]. Furthermore, it is now clear that circRNAs play key roles in diseases correlated with miRNAs [14]. A previous report [15] proposed that an evolutionarily conserved network that regulates EGFR-induced miR-7 expression may target ERF to modulate cell growth in human lung cancer.

As the incidence and mortality rate of GC rank in the top 10 of all cancers in China [16], the diagnosis and treatment GC should receive a great deal of attention. It is well known that the survival rate of GC patients partially depends on the stage of their cancer at diagnosis [16]. In recent decades, certain non-invasive diagnostic techniques such as PET/CT and CTCs (circulating tumor cells) have been increasing accepted for use by clinical doctors. However, because the sensitivity of PET/CT in detecting primary cancers ranges from 21 to 100% [17], and also its high financial cost, the use of PET/CT remains controversial. As CTCs, we need to develop better methods for identifying and isolating subpopulations of tumor cells with down-regulated expression of epithelial proteins [18]. Meanwhile, there is no significant difference in the positive rate of MG7 antigen expression between patients who are *Helicobacter pylori*-negative and *Helicobacter pylori*-positive and have intestinal metaplasia atrophic gastritis or dysplasia of the gastric epithelium. [19]. Li et al. [20] recently described a novel class of stable RNA species in exosomes that may distinguish patients with cancer from healthy control subjects, suggesting the significant potential of that RNA species as a circulating biomarker for cancer diagnosis.

In the present study, we first confirmed that the circLMTK2 was generated from the back-spliced Exon 10 and Exon 11 of the LMTK2 gene located at chromosomal region 7q21.3, and that the splice length of the circLMTK2 was 3109 nucleotides. We found circLMTK2 to be a stable circular RNA expressed in GC cells after treatment with actinomycin D, and that it preferentially localized in the cytoplasm. The above findings suggest that unlike other circRNAs that are considered to be of low abundance or only represent errors in splicing, circLMTK2 might be a non-coding molecule with regulatory functions.

A comparative analysis of circLMTK2 expression in 111 paired GC and normal tissues was performed to better characterize the potential role of circLMTK2 as a biomarker in GC. We found that circLMTK2 expression was significantly lower in GC tissues, and similar results were obtained with human-derived gastric cell lines *in vitro*. Further investigation indicated that circLMTK2 overexpression would effectively inhibit the malignant biological properties of gastric cell line. These results indicated that circLMTK2 might act as a tumor suppressor in GC.

Furthermore, the associative analysis of circLMTK2 expression with the OS of GC patients suggested that a higher level of circLMTK2 expression was correlated with decreased OS rates in GC patients. More importantly, after adjusting for traditional clinical parameters, circLMTK2 remained as an independent risk factor associated with the OS of GC patients, suggesting that circLMTK2 could be used as a prognostic factor in cases of GC.

## Conclusions

Our study first demonstrated that circLMTK2 is down-regulated in gastric cancer compared with adjacent normal tissues, and patients with lower expression of circLMTK2 presented a poor prognosis and survival. Afterward, results from *in vivo* experiments also evidence that overexpression would negatively mediate cancer cells viability and mobility. The present study suggested that circLMTK2 can serve as novel biomarker for predicting a prognosis.

## Abbreviations

GC: gastric cancer
qRT-PCR: quantitative reverse transcription-polymerase chain reaction
